# Global, regional, and national burden and attributable risk factors of neurological disorders: The Global Burden of Disease study 1990–2019

**DOI:** 10.3389/fpubh.2022.952161

**Published:** 2022-11-29

**Authors:** Chenyu Ding, Yuying Wu, Xiaoyong Chen, Yue Chen, Zanyi Wu, Zhangya Lin, Dezhi Kang, Wenhua Fang, Fa Chen

**Affiliations:** ^1^Department of Neurosurgery, Neurosurgery Research Institute, The First Affiliated Hospital, Fujian Medical University, Fuzhou, China; ^2^Fujian Provincial Institutes of Brain Disorders and Brain Sciences, The First Affiliated Hospital, Fujian Medical University, Fuzhou, China; ^3^Department of Neurosurgery, National Regional Medical Center, Binhai Campus of the First Affiliated Hospital, Fujian Medical University, Fuzhou, China; ^4^Department of Epidemiology and Health Statistics, School of Public Health, Fujian Medical University, Fuzhou, China

**Keywords:** neurological disorders, global burden, socio-demographic index, risk factors, DALYs

## Abstract

**Background:**

Neurological disorders are a major and increasing global health challenge, which accounts for a substantial portion of the disease burden worldwide. The aim of this systematic analysis is to present the most comprehensive and up-to-date estimates of disease burden, epidemiological trends, and attributable risk factors of neurological disorders at global, regional, and national levels.

**Methods:**

We extracted data of 18 neurological disorders from the Global Burden of Disease 2019 study database. The burden of neurological disorders was measured using the incidence, prevalence, mortality, and disability-adjusted life years (DALYs), and further described according to age, sex, year, geographical location and socio-demographic Index (SDI). All estimates were presented with corresponding 95% uncertainty intervals (UIs).

**Findings:**

Globally, in 2019, there were nearly 10 million deaths and 349 million DALYs due to neurological disorders. Among the 18 neurological disorders, stroke was the biggest contributor to DALYs (143232.18 [95%UI 133095.81-153241.82] in thousands) and deaths (6552.72 [95%UI 5995.20-7015.14] in thousands), followed by neonatal encephalopathy due to birth asphyxia and trauma. From 1990 to 2019, the DALYs of neurological diseases belonging to the communicable, maternal, neonatal and nutritional categories showed a sharp decrease, while Alzheimer's disease and other dementias and Parkinson's disease showed a large increase. Neurological disorders exhibited different profiles in different regions and age groups. A significant correlation between the SDI and the age-standardized DALY rates was also found except for Alzheimer's disease and other dementias. In addition, risk factors such as high systolic blood pressure, low birth weight and short gestation period, and metabolic risk contribute significantly to neurological disorders.

**Interpretation:**

The overall burden of neurological disorders has increased from 1990 to 2019, especially for non-communicable neurological disorders. The substantial variations of burden across regions emphasize the need for region-specific interventional strategies and allocation of resources based on priorities.

## Introduction

Neurological disorders are a major and increasing global health challenge, which would bring enormous impairment to cognitive-motor function. Mechanistically, neurological disorders could impair cognitive-motor function according to white matter damage, abnormal amyloid deposition, disruption of the blood-brain barrier, synaptic plasticity damage, and impairment of nerve conduction. With a growing population and aging society, more people are reaching ages at which neurological diseases are highly prevalent, contributing substantially to the global disease burden among them, stroke is the leading cause of disability and mortality, resulting in enormous human and economic burdens (1). Over the past dozen years, the absolute number of people disturbed by Parkinson's disease and dementia has more than doubled worldwide (1–3). In addition, the long-neglected disorder, migraine, has climbed to the second largest cause of age-standardized disability-adjusted life-years (DALY) rates for all neurological disorders in the Global Burden of Diseases, Injuries, and Risk Factors Study (GBD) 2016 (1).

Cross-country and cross-region comparisons based on global data could help to better understand the heterogeneous distribution, course, and consequences of these complex diseases. GBD 2016 emphasized the importance of neurological disorders (stroke, migraine, Alzheimer's disease and other dementias. Meningitis, epilepsy, spinal cord injury, brain and other central nervous system cancer, tension-type headache, encephalitis, Parkinson's disease, other neurological disorders, tetanus, multiple sclerosis, motor neuron diseases) by comprehensively estimating their global, regional, and national burden (1). However, the analysis did not include neonatal encephalopathy due to birth asphyxia and trauma, neural tube defects, and down syndrome, which are also significant parts of the global disease burden.

To develop public health plans and improve outcomes for people with these complex and multi-factorial diseases, we need to know the number, and distribution of patients, and their degree of burden (mortality and disability). Additionally, a comprehensive understanding of possible explanations of the heterogeneous course and consequences with effective interventions may contribute to delaying disease onset, and reducing mortality and disability. In this systematic analysis, we presented global, regional, and national accounts and estimated burden of 18 individual neurological disorders by prevalence, incidence, mortality, and DALYs, as well as their trends from 1990 to 2019 by age, sex, and socio-demographic Index (SDI), and their attributable risk factors from updated surveys, which could contribute to guiding policy formulation and improving the allocation of limited health care resources.

## Methods

### Overview

Data on the burden of neurological disorders was extracted from the GBD 2019 *via* the Global Health Data Exchange website (http://ghdx.healthdata.org). The GBD 2019 was created by GBD collaborators, which systematically assessed the health burden of 369 diseases and injuries in 204 countries and territories from 1990 to 2019. The detailed methodologies for estimating incidence, prevalence, deaths and DALYs have been described elsewhere (4, 5). This study was compliant with the Guidelines for Accurate and Transparent Health Estimates Reporting.

### Definition of neurological disorders

In this study, 18 neurological disorders were divided into three categories: non-communicable diseases (stroke, migraine, Alzheimer's disease and other dementias, idiopathic epilepsy, brain and central nervous system cancer, neural tube defects, Parkinson's disease, tension-type headache, down syndrome, multiple sclerosis, motor neuron disease, and other neurological disorders); communicable, maternal, neonatal and nutritional diseases (neonatal encephalopathy due to birth asphyxia and trauma, meningitis, encephalitis, tetanus); injuries (head Injuries, and spinal Injuries). The International Classification of Diseases ninth and tenth revision (ICD-9 and ICD-10) codes used in the analyses for neurological disorders were summarized in Supplementary Table S1.

### Data processing and presentation

GBD 2019 modeling strategies for estimating cause-specific deaths and DALYs have been described in detail elsewhere (4, 5). We described the burden of neurological disorders according to different years, sex, age, SDI regions, and geographical locations. SDI was evaluated based on the total fertility rate among females younger than 25 years old, educational attainment for those aged 15 years or older, and lag distributed income per capita. The 204 countries and territories were then placed in 5 categories according to the SDI: low-SDI, low-middle-SDI, middle-SDI, high-middle-SDI, and high-SDI.

In GBD 2019, every estimate was calculated 1,000 times, and the final data were presented as the mean of these estimates. 95% uncertainty interval (UI) was calculated for each parameter in the analysis, which was determined using the 25 and 975th values of the ordered 1,000 draws. The Pearson correlation coefficient was used to analyze the association of SDI and DALYs and prevalence of 18 neurological disorders. We performed Joinpoint regression models to evaluate the temporal trends in age-standardized death and DALY rates attributable to risk factors. Specifically, line segments were drawn on a logarithmic scale to describe the trend, the “joinpoints” of these lines were connected, and points where the trend linear slope significantly changed over time were identified. This regression was performed using the Joinpoint software (version 4.7.0) developed by the Surveillance Research Program of the US National Cancer Institute.

## Results

### Overall burden

**Figure 1:**
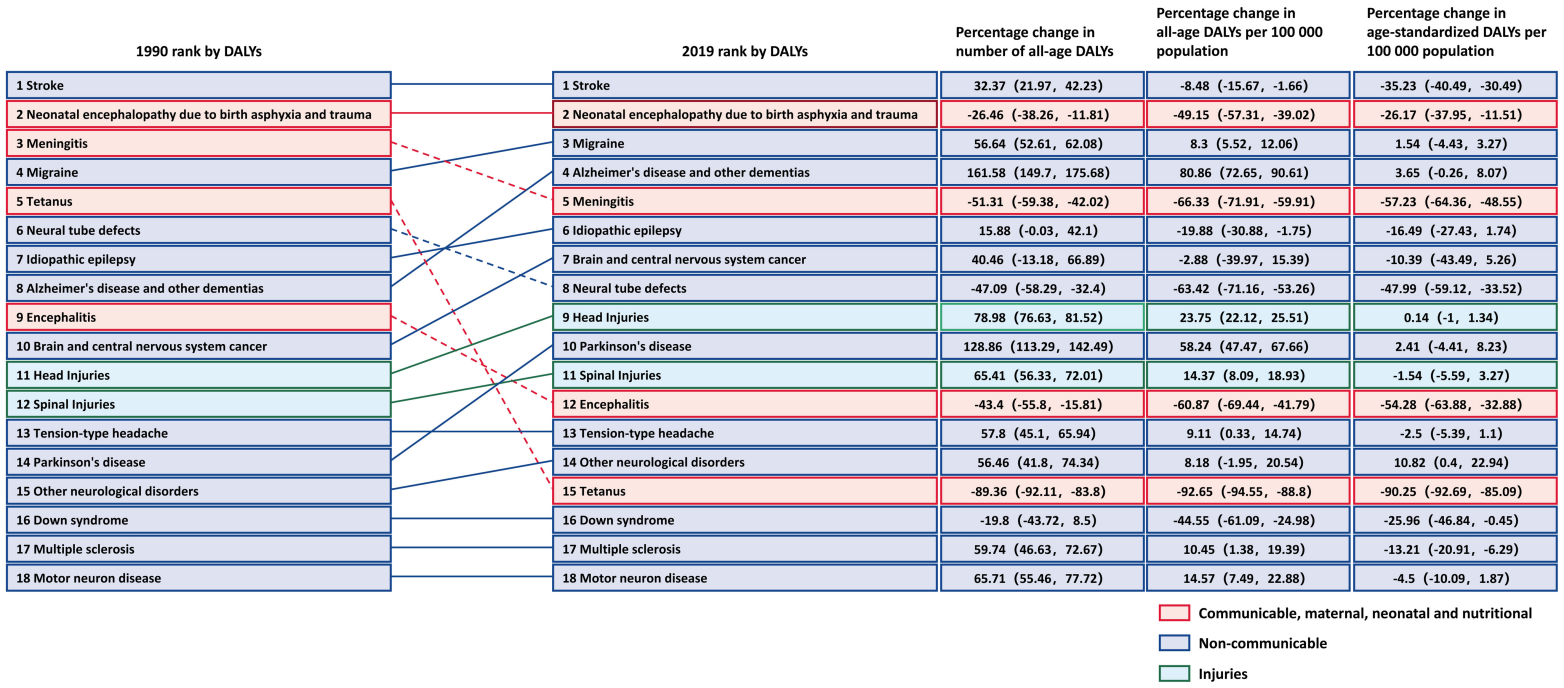
Changes in rank and percentage of global DALYs, in terms of total DALYs, percentage change in numbers of DALYs and age-standardized DALY rates due to 18 neurological disorders for all ages and both sexes from 1990 to 2019. Solid lines indicate an increase in rank while dashed lines represent a decrease in rank.

Globally, these 18 major neurological disorders resulted in 10.06 million deaths and 349.22 million DALYs in 2019. When combined, these neurological disorders were the main cause of the 2019 global DALYs, only after cardiovascular diseases (stroke not included). The incidence, prevalence, DALYs, and deaths associated with each neurological disorder were summarized in Table 1. In 2019, of these neurological disorders, stroke accounted for the highest deaths and DALYs, followed by neonatal encephalopathy due to birth asphyxia and trauma. Of note, although migraine was not one of the leading causes of death, it was the third leading cause of DALYs (Figure 1).

**Table 1:** Global deaths, DALYs, incidence, and prevalence and age-standardized rates by neurological disorders, 1990–2019.

	**Absolute number, in thousand (95% UI)**		**Age-standardized rate (95% UI)**			
	**1990**	**2019**	**1990**	**2019**		
				**Both**	**Male**	**Female**
**Non-communicable**						
**Stroke**						
Deaths	4,574.08 (4,295.93, 4,892.81)	6,552.72 (5,995.20, 7,015.14)	132.44 (123.08, 141.7)	84.19 (76.76, 90.15)	96.36 (87.63, 104.21)	73.5 (65.21, 80.66)
DALYs	108,206.65 (102,415.59, 114,799.15)	143,232.18 (133,095.81, 153,241.82)	2,729.87 (2,579.92, 2,901.17)	1,768.05 (1,640.65, 1,889.39)	2,024.28 (1,852.42, 2,195.62)	1,531.27 (1,397.07, 1,667.6)
Incidence	7,186.71 (6,562.19, 7,939.71)	12,224.55 (11,041.82, 13,589.31)	181.4 (165.21, 199.76)	150.77 (136.52, 167.46)	151.1 (136.9, 167.54)	149.75 (135.58, 166.56)
Prevalence	54,747.92 (50,221.31, 59,562.11)	101,474.56 (93,211.91, 110,526.30)	1,320.79 (1,212.44, 1,440.5)	1,240.26 (1,139.71, 1,352.99)	1,150.19 (1,052.72, 1,259.3)	1,316.73 (1,210.72, 1,433.71)
**Migraine**						
DALYs	26,863.35 (3,969.24, 61,445.23)	42,077.67 (6,418.38, 95,645.21)	517.58 (81.95, 1,169.12)	525.54 (78.79, 1,193.99)	389.82 (65.32, 874.29)	662.26 (92.04, 1,522.56)
Incidence	62,585.28 (54,459.80, 70,979.26)	87,648.97 (76,635.69, 98,654.60)	1,119.53 (977.26, 1,262.34)	1,142.54 (995.9, 1,289.44)	853.29 (738.29, 972.46)	1,440.66 (1,258.51, 1,621.51)
Prevalence	721903.03 (624861.19, 833350.78)	1128076.26(979598.83.1298138.08)	13865.65 (12040.68, 15907.49)	14107.26 (12270.27, 16239.02)	10337.63 (8947.99, 12013)	17902.51 (15588.26, 20531.74)
**Alzheimer's disease and other dementias**						
Deaths	560.93 (135.27, 1,546.92)	1,623.28 (407.47, 4,205.72)	22.24 (5.5, 59.98)	22.92 (5.83, 59.2)	20.71 (5.03, 55.21)	24.19 (6.24, 61.53)
DALYs	9,663.31 (4,233.95, 21,374.78)	25,276.99 (11,204.52, 54,558.24)	326.71 (143.33, 731.03)	338.64 (151.02, 731.27)	304.47 (130.97, 679.76)	361.2 (162.8, 767.72)
Incidence	2,920.98 (2,485.35, 3,372.86)	7,236.38 (6,217.24, 8,232.67)	93.58 (80.12, 106.7)	94.99 (81.59, 107.86)	83.67 (71.06, 95.65)	103.45 (89.24, 117.15)
Prevalence	19,788.20 (16,933.47, 22,794.38)	51,624.19 (44,276.97, 59,021.50)	645.89 (552.86, 743.62)	682.48 (585.2, 782.73)	585.87 (497.37, 672.86)	748.15 (646.36, 856.85)
**Idiopathic epilepsy**						
Deaths	100.05 (81.18, 112.23)	114.01 (100.18, 129.93)	1.94 (1.61, 2.15)	1.46 (1.28, 1.67)	1.71 (1.53, 1.98)	1.21 (0.96, 1.39)
DALYs	11,285.62 (8,614.05, 14,136.60)	13,077.62 (9,986.73, 16,734.09)	204.32 (157.63, 254.14)	170.63 (130.42, 218.26)	187.41 (145.35, 237.74)	153.81 (115.82, 197.91)
Incidence	1,859.52 (1,281.78, 2,526.09)	2,898.22 (2,098.72, 3,823.38)	33.22 (23.4, 44.69)	38.82 (27.99, 51.28)	41.83 (30.36, 55.18)	35.78 (25.63, 47.63)
Prevalence	15,324.10 (11,446.73, 19,630.58)	25,111.11 (19,033.57, 31,433.01)	288.79 (218.16, 364.76)	326.27 (247.83, 408.33)	340.47 (259.56, 425.59)	312.43 (236.28, 393.98)
**Brain and central nervous system cancer**						
Deaths	139.63 (119.90, 182.29)	246.25 (185.64, 270.93)	3.08 (2.71, 4.01)	3.05 (2.29, 3.36)	3.58 (2.55, 4.05)	2.56 (1.82, 2.88)
DALYs	6,165.30 (4,958.44, 8,491.43)	8,659.87 (6,718.03, 9,574.46)	121.67 (100.03, 164.94)	109.04 (84.57, 120.92)	127.56 (92.92, 146.21)	90.85 (67.8, 102.01)
Incidence	179.05 (152.53, 237.07)	347.99 (262.08, 388.90)	3.82 (3.34, 5)	4.34 (3.27, 4.86)	4.84 (3.49, 5.56)	3.89 (2.78, 4.46)
Prevalence	423.57 (360.09, 559.07)	1,065.29 (800.44, 1,199.91)	8.44 (7.3, 10.94)	13.48 (10.09, 15.19)	13.47 (9.68, 15.48)	13.54 (9.79, 15.72)
**Neural tube defects**						
Deaths	164.26 (116.63, 263.47)	85.06 (62.61, 121.83)	2.54 (1.8, 4.08)	1.29 (0.95, 1.85)	1.21 (0.77, 1.92)	1.38 (0.94, 2.08)
DALYs	14,634.99 (10,465.48, 23,313.44)	7,743.43 (5,726.20, 11,022.80)	226.09 (161.47, 360.55)	117.59 (86.95, 167.2)	109.9 (71.12, 171.49)	125.81 (86.67, 186.62)
Incidence	178.69 (138.87, 225.52)	136.01 (108.11, 169.88)	2.72 (2.11, 3.43)	2.1 (1.67, 2.62)	1.89 (1.5, 2.37)	2.32 (1.85, 2.89)
Prevalence	827.38 (671.36, 1,020.25)	1,094.32 (888.06, 1,335.86)	13.86 (11.25, 17)	15.04 (12.17, 18.36)	13.8 (11.2, 16.95)	16.34 (13.19, 19.97)
**Parkinson's disease**						
Deaths	147.24 (137.33, 158.06)	362.91 (326.85, 388.20)	4.62 (4.28, 4.94)	4.79 (4.3, 5.13)	6.66 (6.02, 7.24)	3.51 (3.04, 3.82)
DALYs	2,749.55 (2,541.53, 2,992.89)	6,292.62 (5,769.21, 6,827.21)	78.09 (72.26, 84.66)	79.97 (73.25, 86.6)	106.44 (97.18, 115.48)	59.91 (53.22, 65.43)
Incidence	416.47 (362.67, 473.21)	1,081.72 (953.26, 1,211.20)	11.22 (9.87, 12.68)	13.43 (11.84, 15.02)	17.79 (15.75, 19.7)	9.95 (8.77, 11.18)
Prevalence	3331.16 (2834.58, 3886.67)	8,511.02 (7,288.53, 9,841.38)	91.74 (78.27, 106.72)	106.28 (91.2, 122.21)	129.33 (111.27, 149.39)	87.59 (75.33, 101.2)
**Tension-type headache**						
DALYs	2,878.10 (853.08, 9,769.27)	4,541.69 (1,395.55, 14,981.34)	57.65 (17.73, 188.5)	56.21 (17, 188.51)	49.99 (14.07, 181.96)	62.37 (19.79, 186.62)
Incidence	472,024.83 (416,622.25, 527,313.85)	706,190.11 (626,723.55, 788,575.30)	9,006.97 (7,976.7, 10,044.71)	8,968.18 (7,931.86, 9,990.52)	8,739.42 (7,724.11, 9,757.24)	9,193.12 (8,138.66, 10,255.54)
Prevalence	1,307,510.96 (1,142,071.27, 1,483,958.17)	1995172.55 (1751946.85, 2242204.89)	25,306.23 (22,263.45, 28,510.58)	25,113.49 (22,020.81, 28,316.24)	24,370.27 (21,275.15, 27,422.94)	25,839.52 (22,688.09, 29,083.27)
**Down syndrome**						
Deaths	25.38 (16.90, 52.83)	22.28 (17.76, 33.17)	0.41 (0.27, 0.84)	0.32 (0.25, 0.48)	0.31 (0.25, 0.53)	0.32 (0.22, 0.53)
DALYs	2,223.90 (1,499.51, 4,609.11)	1,783.57 (1,374.19, 2,747.16)	35.14 (23.81, 72.35)	26.02 (19.83, 40.75)	25.74 (20.22, 45.11)	26.33 (18.11, 44.13)
Incidence	80.06 (61.96, 102.45)	78.43 (60.13, 101.73)	1.22 (0.94, 1.56)	1.21 (0.93, 1.57)	1.27 (0.98, 1.65)	1.15 (0.88, 1.49)
Prevalence	1,257.11 (989.42, 1,573.67)	1,579.78 (1,251.95, 1,962.09)	21.18 (16.71, 26.54)	21.51 (16.98, 26.79)	23.37 (18.47, 29.27)	19.53 (15.48, 24.24)
**Multiple sclerosis**						
Deaths	13.36 (11.90, 17.57)	22.44 (20.23, 27.79)	0.32 (0.28, 0.42)	0.27 (0.24, 0.34)	0.23 (0.19, 0.31)	0.31 (0.25, 0.39)
DALYs	726.07 (621.89, 867.80)	1,159.83 (1,001.18, 1,381.87)	16.09 (13.8, 19.34)	13.96 (12.05, 16.63)	10.84 (9.25, 13.57)	16.98 (14.32, 20.53)
Incidence	41.85 (36.31, 47.44)	59.35 (51.82, 66.94)	0.8 (0.7, 0.9)	0.74 (0.65, 0.83)	0.55 (0.48, 0.63)	0.93 (0.82, 1.05)
Prevalence	1,022.94 (874.53, 1,181.20)	1,756.79 (1,531.92, 1,973.62)	22.65 (19.45, 26.02)	21.25 (18.52, 23.91)	14.13 (12.21, 16.06)	28.08 (24.56, 31.54)
**Motor neuron disease**						
Deaths	17.65 (17.01, 18.27)	39.08 (36.57, 41.13)	0.43 (0.41, 0.44)	0.48 (0.45, 0.51)	0.57 (0.53, 0.6)	0.4 (0.37, 0.43)
DALYs	624.36 (594.25, 665.30)	1,034.61 (979.91, 1,085.40)	13.25 (12.7, 13.92)	12.66 (11.98, 13.29)	14.89 (13.97, 15.68)	10.59 (9.92, 11.22)
Incidence	35.59 (31.62, 40.07)	63.70 (57.30, 71.34)	0.79 (0.71, 0.89)	0.79 (0.72, 0.88)	0.9 (0.82, 1)	0.7 (0.62, 0.78)
Prevalence	159.07 (134.17, 187.02)	268.67 (231.89, 310.66)	3.31 (2.83, 3.85)	3.37 (2.9, 3.87)	3.72 (3.23, 4.26)	3.05 (2.62, 3.54)
**Other neurological disorders**						
Deaths	34.10 (31.53, 36.60)	59.61 (55.08, 64.55)	0.76 (0.71, 0.81)	0.76 (0.7, 0.82)	0.88 (0.81, 0.96)	0.65 (0.59, 0.73)
DALYs	2,724.96 (2,242.50, 3,282.80)	4,263.39 (3,458.86, 5,174.14)	50.48 (42.48, 60.27)	55.94 (45.19, 68.29)	61.23 (50.08, 74.43)	50.65 (39.88, 63.26)
Prevalence	34.20 (23.20, 46.94)	56.88 (39.07, 77.83)	0.68 (0.46, 0.94)	0.72 (0.49, 0.99)	0.8 (0.55, 1.08)	0.65 (0.44, 0.89)
**Communicable, maternal, neonatal and nutritional**						
**Neonatal encephalopathy due to birth asphyxia and trauma**						
Deaths	808.68 (726.80, 903.20)	566.98 (475.54, 672.55)	12.31 (11.07, 13.75)	8.75 (7.34, 10.38)	9.87 (8.02, 12)	7.56 (6.39, 8.88)
DALYs	72,555.08 (65,080.04, 81,070.34)	53,356.61 (45,101.60, 62,832.90)	1,106.6 (992.57, 1,236.27)	817.02 (691.13, 964.38)	917.65 (755.28, 1,101.42)	709.27 (607.04, 829.12)
Incidence	1,335.11 (907.23, 2,086.32)	1,378.70 (963.70, 2,024.06)	20.3 (13.79, 31.72)	21.29 (14.88, 31.25)	22.16 (15.47, 32.8)	20.35 (14.35, 29.3)
Prevalence	3,180.38 (2,109.70, 4,882.93)	10,567.81 (8,145.65, 13,330.69)	56.73 (37.73, 86.72)	140.45 (108.38, 177.4)	143.75 (112.12, 179.83)	136.94 (105.57, 174.36)
**Meningitis**						
Deaths	432.52 (376.42, 494.07)	236.22 (204.38, 277.43)	7.47 (6.58, 8.45)	3.29 (2.82, 3.89)	3.56 (3.05, 4.27)	3.01 (2.53, 3.57)
DALYs	33,545.07 (28,880.19, 38,724.98)	16,333.20 (13,775.12, 19,609.77)	547.17 (472.56, 628.53)	234.01 (195.83, 282.54)	251.51 (209.74, 308.95)	215.73 (176.96, 262.54)
Incidence	3,288.78 (2,698.47, 4,000.59)	2,507.22 (2,113.07, 2,988.70)	55.27 (45.94, 66.67)	35.42 (29.57, 42.47)	36.84 (30.66, 44.3)	33.94 (28.38, 40.56)
Prevalence	10,072.12 (8,467.41, 12,082.02)	7,683.54 (6,590.29, 9,132.20)	189.23 (159.48, 226.44)	99.88 (85.55, 118.79)	101.02 (86.3, 120.13)	98.77 (84.82, 117.17)
**Encephalitis**						
Deaths	117.80 (95.32, 138.25)	89.90 (76.53, 122.87)	2.18 (1.79, 2.51)	1.19 (1.01, 1.62)	1.29 (1.08, 1.9)	1.09 (0.88, 1.56)
DALYs	8,476.49 (6,719.34, 10,268.57)	4,797.41 (4,059.49, 6,418.09)	142.88 (115.16, 171.01)	65.32 (55.05, 87.29)	69.8 (57.66, 98.46)	60.83 (49.96, 86.29)
Incidence	1,284.16 (1,114.30, 1,475.91)	1,444.72 (1,280.15, 1,614.94)	23.17 (20.34, 26.27)	19.33 (17.06, 21.7)	20.15 (17.8, 22.59)	18.62 (16.42, 20.93)
Prevalence	4,297.36 (3,137.49, 5,414.77)	4,499.43 (3,372.09, 5,573.17)	83.33 (60.28, 105.14)	56.78 (42.65, 70.29)	57.22 (42.6, 71.13)	56.44 (42.78, 69.62)
**Tetanus**						
Deaths	275.38 (240.55, 322.38)	34.68 (25.94, 48.46)	4.61 (4.02, 5.43)	0.49 (0.37, 0.68)	0.54 (0.38, 0.8)	0.44 (0.32, 0.61)
DALYs	21,780.38 (18,499.61, 25,499.24)	2,316.38 (1,770.00, 3,279.41)	345.97 (297.35, 405.79)	33.74 (25.57, 47.85)	36.31 (26.69, 55.68)	31.1 (22.97, 44.41)
Incidence	615.73 (487.72, 778.30)	73.66 (53.35, 101.15)	10.26 (8.2, 12.85)	1.03 (0.74, 1.42)	1.15 (0.78, 1.66)	0.9 (0.65, 1.28)
Prevalence	172.99 (135.12, 219.36)	61.66 (44.60, 81.24)	3.1 (2.41, 3.93)	0.81 (0.59, 1.07)	0.81 (0.59, 1.06)	0.82 (0.59, 1.08)
**Injuries**						
**Head Injuries**						
DALYs	3,953.93 (5,334.85, 2,777.81)	7,076.9 (9,588.1, 4,997.95)	86.4 (60.64, 116.54)	86.52 (61.14, 117.19)	109.13 (77.17, 148.05)	63.96 (45.25, 86.36)
Incidence	18,853.87 (16,215.39, 21,806.11)	27,160.71 (23,357.25, 31,415.05)	366.11 (316.59, 421.11)	345.86 (297.81, 401.02)	431.04 (373.37, 496.38)	257.51 (219.22, 301.35)
Prevalence	27,223.25 (26,109.47, 28,451.59)	48,987.93 (46,840.15, 51,316.8)	599.34 (575.82, 626.02)	599.26 (573.04, 627.34)	749.82 (715.64, 787.62)	449.11 (429.79, 469.55)
**Spinal injuries**						
DALYs	3,748.67 (4,951.85, 2,702.78)	6,200.81 (8,156.19, 4,465.32)	77.37 (56.13, 100.8)	76.18 (54.77, 100.37)	87.32 (62.62, 113.86)	64.66 (46.47, 87.36)
Incidence	595.23 (471.5, 766.66)	909 (706.95, 1,156.41)	12.28 (9.74, 15.61)	11.53 (8.97, 14.67)	12.58 (10.04, 15.78)	10.27 (7.8, 13.45)
Prevalence	11,366.8 (10,375.24, 13,112.53)	20,635.04 (18,925.88, 23,611.17)	239.15 (220.1, 272.16)	253.08 (231.49, 290.37)	284.3 (261.07, 321.91)	220.26 (201.08, 257.27)

**Figure 2:**
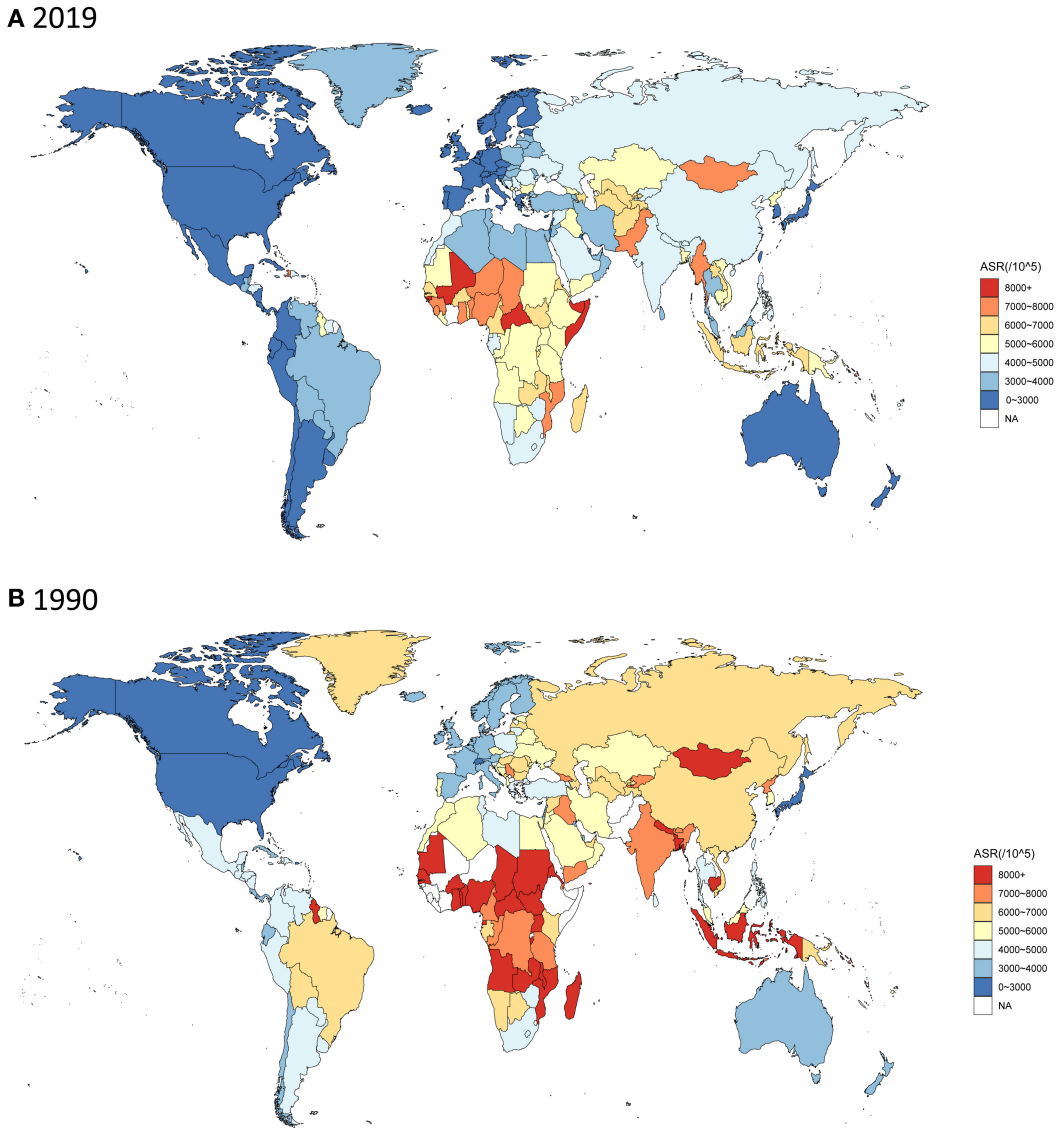
Age-standardized DALY rates of neurological disorders for all ages and both sexes among 204 countries and territories. **(A)** in 2019; **(B)** in 1990.

**Figure 3:**
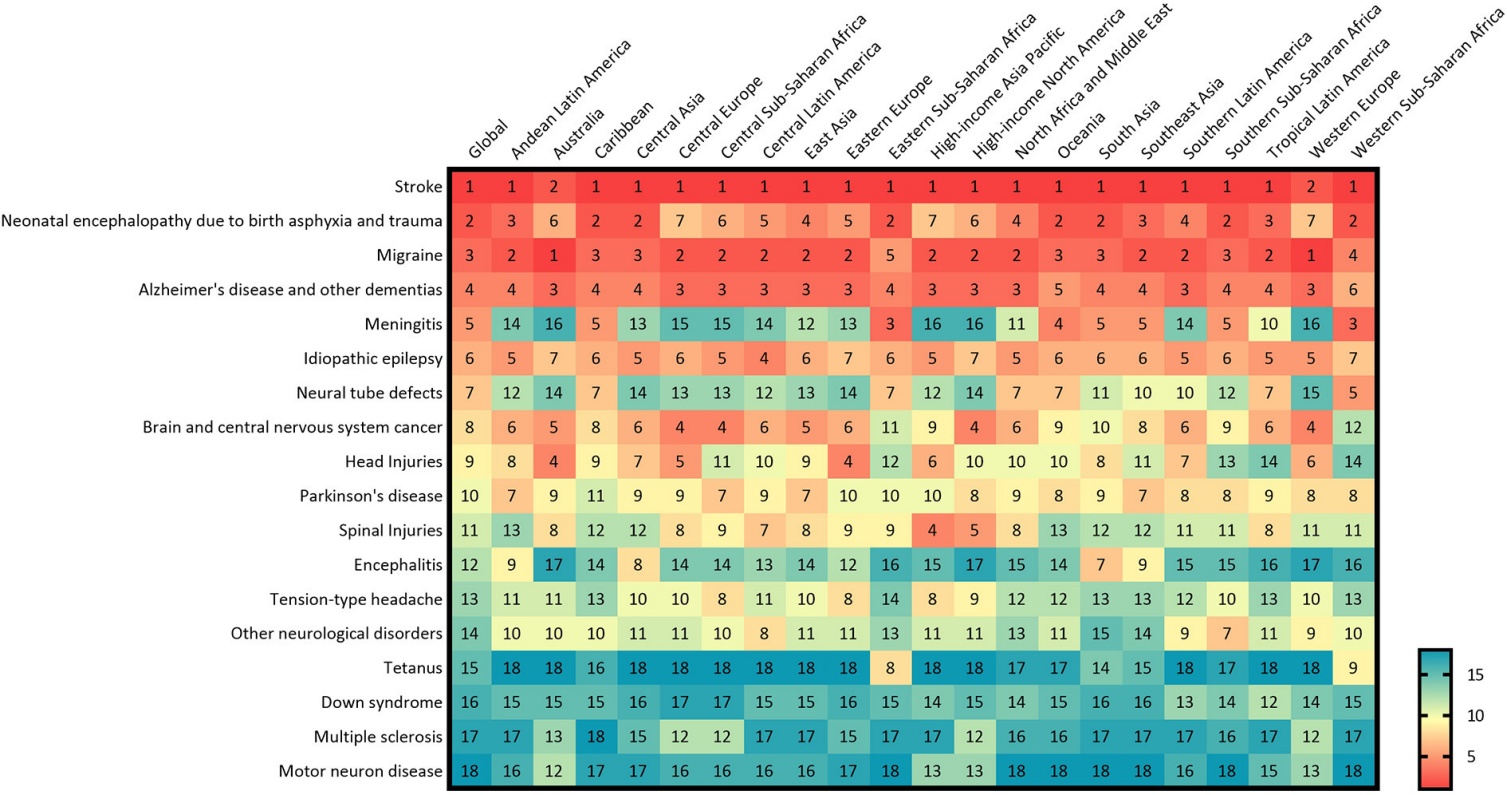
Ranking of age-standardized DALY rates for neurological disorders by 21 GBD regions, for both sexes, 2019.

High age-standardized DALY rates of these neurological disorders were observed in Western, Central, and Eastern Africa, most countries in East and Southeast Asia, of which most are coastal countries. In contrast, these rates were low in America, Europe and Oceania (Figure 2B). Among specific countries, Mali, Central African Republic, Somalia, Kiribati, Solomon Islands and Guinea-Bissau showed the greatest burden (DALYs >8000/100, 000 population) (Supplementary Table S2). From 1990 to 2019, most regions, including South America, Asia, Malay Archipelago, and most territories of Central Africa, showed a significant decrease in DALYs (Figure 2). Stroke, migraine, Alzheimer's disease and other dementias were the major causes of neurological disease burden of the 21 GBD regions. Some diseases showed significantly different trends in different countries and territories (Figure 3).

### Stroke

**Figure 4:**
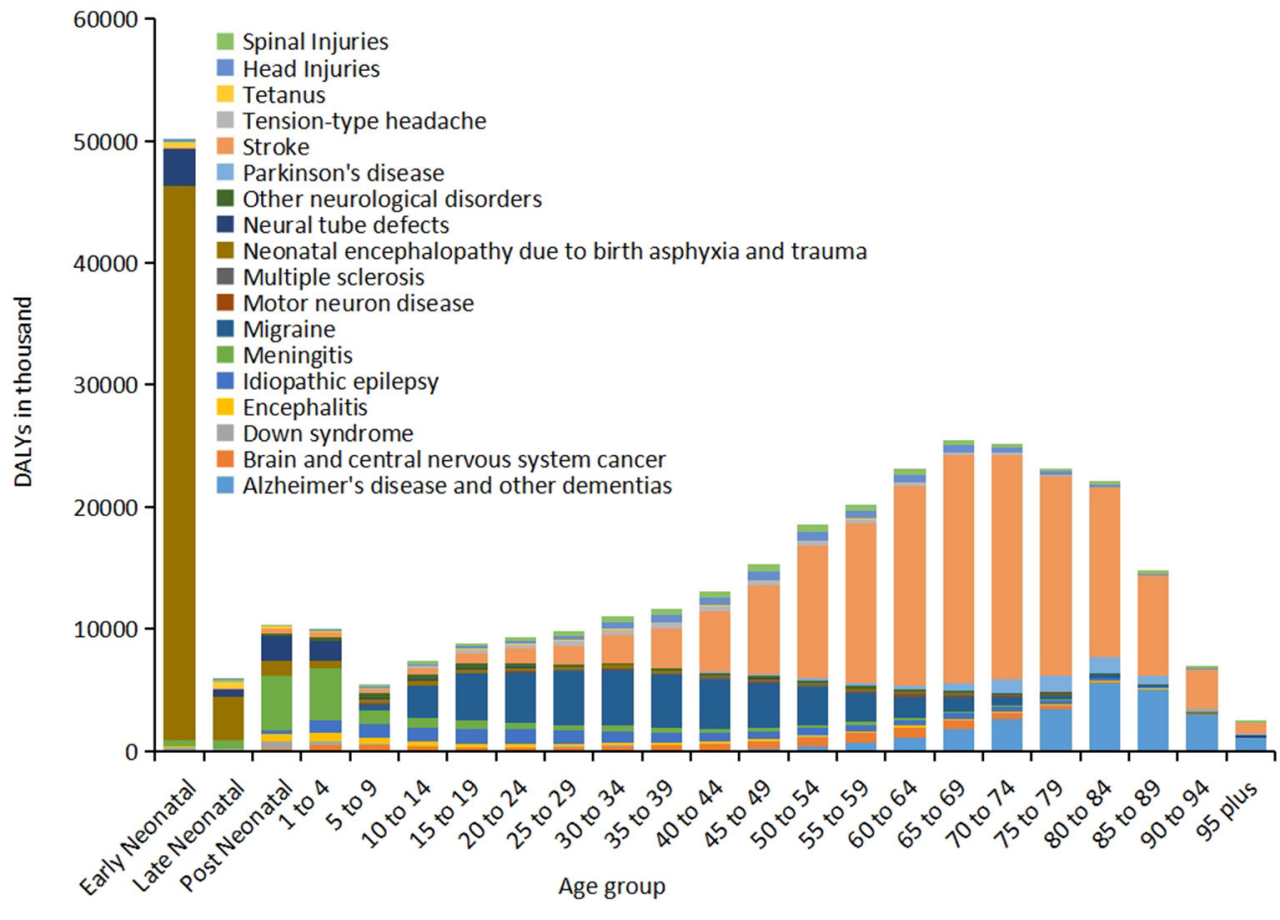
Distribution of global DALYs for 18 neurological disorders by age, 2019.

In 2019, there were 12,224.55 (95%UI 11,041.82–13,589.31) thousands incidence of stroke, causing 6,552.72 (95%UI 5,995.20–7,015.14) thousands deaths, and 143,232.18 thousands (95%UI 133,095.81–153,241.82) DALYs worldwide (Table 1). Among the 18 neurological disorders, DALYs due to stroke ranked first. From 1990 to 2019, although number of DALY due to stroke increased by 32.37% (95%UI 21.97–42.23), percentage change in age-standardized DALY rate (per 100,000 population) decreased by 35.23% (95%UI 30.49–40.49) (Figure 1). The age-standardized DALY rate of stroke was highest in Solomon Islands, followed by Kiribati and Nauru (Supplementary Table S2). In 21 GBD regions, stroke was the leading cause of disease burden in 19 regions (Figure 3). Age-standardized DALY rate showed a moderate negative correlation with the SDI (*r* = −0.50, *p* < 0.001) (Supplementary Table S3). In 2019, globally, stroke was the leading cause of the DALYs in neurological disorders for the population 50 years or older, and peaked for the 65–74 years old age group (Figure 4).

### Neonatal encephalopathy due to birth asphyxia and trauma

As the same in 1990, in 2019, of the 18 neurological disorders, neonatal encephalopathy due to birth asphyxia and trauma was still the second cause of disease burden, only after the stroke (Figure 1), with age-standardized DALY and death rates (per 100,000 population) being 817.02 (95%UI 691.13–964.38) and 8.75 (95%UI 7.34–10.38), respectively (Table 1). From 1990 to 2019, although percentage change in the number and age-standardized DALY rate showed an overall decreasing trend, the age-standardized prevalence rate increased by 150.26% (221.57, 91.72) (Figure 1). The disease burden showed significant regional differences, and ranked in the top three leading in most regions (10/21) (Figure 3). Age-standardized DALY rate showed a strong negative correlation with the SDI (*r* = −0.78, *p* < 0.001) (Supplementary Table S3). In early neonatal and late neonatal, the DALY rates were mostly attributable to neonatal encephalopathy due to birth asphyxia and trauma, which caused the highest global DALYs of the early neonatal age group (Figure 4).

### Migraine

Compared to 1990, in 2019, the global disease burden of migraine moved from the fourth highest to the third highest, and percentage change in the number of all-age DALYs was 56.64 (95%UI 52.61–62.08) (Figure 1). In 2019, the incidence and prevalence of migraine were 87,648.97 (76,635.69, 98,654.60) and 1,128,087.26 (979,598.83, 1,298,138.08) in thousands, respectively, which only after tension-type headache, but it caused a higher DALYs than tension-type headache did. The incidence, prevalence, and DALYs due to migraine for women were significantly higher than those for men (Table 1). Of note, the age-standardized DALY and prevalence rates were positively correlated with the SDI (r value was 0.22 and 0.20, respectively. *p* < 0.001) (Supplementary Table S3). In terms of age, migraine mainly impacted the young population, resulting in the highest disease burden in the 15–34 years old group. Out of the 18 neurological disorders, it also led to a major disease burden for this age group (Figure 4).

### Alzheimer's disease and other dementias

In 2019, there were 7,236.38 (95%UI 6,217.24–8,232.67) thousands incidence and 51,624.19 (95%UI 44,276.97–59,021.50) thousands prevalence of Alzheimer's disease and other dementias, which caused 25,276.99 (95%UI 11,204.52–54,558.24) DALYs and 1,623.28 (95%UI 407.47–4,205.72) deaths in thousands globally (Table 1). In 1990, the DALYs due to Alzheimer's disease and other dementias ranked the eighth highest, and in 2019, it jumped to the fourth highest (Percentage change was 161.58 (95%UI 149.7–175.68) (Figure 1). The age-standardized prevalence rate showed a moderate positive correlation with the SDI (*r* = 0.50, *p* < 0.001) (Supplementary Table S3). The burden of Alzheimer's disease and other dementias was mainly seen in the elderly. It increased with age, and peaked in the 80–89 years old group (Figure 4).

### Meningitis

From 1990 to 2019, the DALYs due to meningitis moved down from the third highest to the fifth highest. The percentage change in age-standardized DALYs was −57.23 (95%UI −64.36, −48.55) (Figure 1). The age-standardized rates of incidence, prevalence, and deaths also decreased (Table 1). In 21 GBD regions, the burdens of meningitis showed significant differences. In Eastern sub-Saharan Africa and Western sub-Saharan Africa, meningitis ranked the third highest in terms of disease burden out of the 18 neurological disorders (Figure 3). The age-standardized DALY rate showed a strong negative correlation with the SDI (*r* = −0.76, *p* < 0.001) (Supplementary Table S3). In terms of age, meningitis mainly impacted the post-neonatal and 1–4 years old group in terms of disease burden (Figure 4).

### Idiopathic epilepsy

In 2019, the incidence and prevalence of idiopathic epilepsy were 2,898.22 (2.098.72, 3.823.38) in thousands and 25,111.11 (19.033.57, 31.433.01) in thousands, respectively, which resulted in 13,077.62 (9.986.73, 16.734.09) thousands DALYs and 114.01(100.18, 129.93) thousands deaths. From 1990 to 2019, both numbers and age-standardized rates of incidence and prevalence increased, despite this trend, age-standardized rates of DALYs and deaths decreased (Table 1). The age-standardized DALY rate showed a strong negative correlation with the [SDI*r* = −0.68, *p* < 0.001 (Supplementary Table S3)]. In terms of age, idiopathic epilepsy mainly caused disease burden for the 5–30 years old group (Figure 4).

### Neural tube defects

Neural tube defects caused 7,743.43 (95%UI 5,726.20, 11,022.80) thousands DALYs in 2019, which showed a decreasing trend of 47.1% (95%UI 32.40, 58.29) from 1990 to 2019. Crude numbers and age-standardized rates of incidence and deaths also decreased, but the prevalence increased. The burden on neural tube defects showed distinct regional distribution (Table 1 and Figure 1). It ranked the 15th in Western Europe, but ranked the 5th in Western sub-Saharan Africa (Figure 3). Age-standardized DALY rate showed a strong negative correlation with the SDI (*r* = −0.83, *p* < 0.001) (Supplementary Table S3). In terms of age, the disease burden of neural tube defects mainly impacted the early neonatal, post neonatal and 1–4 years old groups (Figure 4).

### Brain and central nervous system cancer

The rank of burden of brain and central nervous system cancer increased from the 10th in 1990 to the 7th in 2019 (Figure 1). In 2019, there were 347.99 (95%UI 262.08–388.90) incidents and 1,065.29 (95%UI 800.44, 1,199.91) prevalence of brain and central nervous system cancer in thousands, which caused 8,659.87 (95%UI 6,718.03–9,574.46) DALYs and 246.25 (95%UI 185.64–270.93) deaths in thousands (Table 1) worldwide. The age-standardized DALY rate showed a moderate positive correlation with the SDI (*r* = 0.53, *p* < 0.001), and age-standardized prevalence rate showed a strong positive correlation with the SDI (*r* = 0.78, *p* < 0.001) (Supplementary Table S3). In terms of age, the burden of brain and central nervous system cancer mainly impacted the 45–69 years old group (Figure 4).

### Head injuries

From 1990 to 2019, the rank of DALYs due to head injuries moved up from the 11th to the 9th, which increased by 23.75% (22.12, 25.51) in all-age DALYs per 100,000 population (Figure 1). The crude numbers of incidence and prevalence also showed a huge increase, despite the relatively stable age-standardized rates (Table 1). In GBD regions, there was a significant regional distribution. In Australia and Eastern Europe, it ranked the 4th, while in Tropical Latin America and Western sub-Saharan Africa, it ranked the 14th (Figure 3). The age-standardized DALY and prevalence rates showed a moderate positive correlation with the SDI (*r* = 0.47, *p* < 0.001) (Supplementary Table S3). In terms of age, the burden of head injuries was mainly seen in the 35–69 years old group, and was higher for males (Figure 4).

### Parkinson's disease

Compared to 1990, DALYs due to Parkinson's disease increased significantly in 2019, with a 128.86% (113.29, 142.49) increase in the number of all-age DALYs, resulting in the rank of disease burden moved up from the 14 to 10th (Figure 1). The burden on Parkinson's disease peaked for the 70–84 years old group, and the DALYs of men was much higher than that of women (Figure 4 and Table 1). Of note, age-standardized prevalence rate showed a moderate positive correlation with the SDI (*r* = 0.53, *p* < 0.001), while age-standardized DALY rate showed a weak negative correlation with the SDI (*r* = −0.21, *p* < 0.001) (Supplementary Table S3).

### Spinal injuries

From 1990 to 2019, the age-standardized DALY, incidence and prevalence rates did not change significantly (Table 1). The disease burden mainly impacted the 30–65 years old group (Figure 4). Both the age-standardized prevalence and DALY rates showed a positive correlation with the SDI (all *p* < 0.001) (Supplementary Table S3), as the disease burden of spinal injuries ranked higher in high SDI regions such as high-income Asia Pacific and high-income North America (Figure 3).

### Encephalitis

In 2019, encephalitis resulted in 4,797.41(4,059.49, 6,418.09) in thousands DALYs (Table 1), which significantly decreased by 54.28% (32.88, 63.88), and lowered its rank from the 9th to the 12th compared to 1990 (Figure 1). In terms of age, the disease burden was mainly seen in the post neonatal and 1–9 years old groups (Figure 4). Andean Latin America, Central Asia, South Asia, and Southeast Asia had higher disease burdens compared to other regions (Figure 3). Similar to meningitis, the age-standardized prevalence and DALY rates showed a negative correlation with the SDI (all *p* < 0.001) (Supplementary Table S3).

### Tension-type headache

In 2019, tension-type headache showed very high incidence (706,190.11 [626,723.55, 788,575.30] in thousands) and prevalence (1,995,172.55 [1,751,946.85, 2,242,204.89] in thousands), and caused 4,541.69(1,395.55, 14,981.34) in thousands DALYs (Table 1), which increased by 57.8% (45.1, 65.94) in the number of all-age DALYs compared to 1990 (Figure 1). The 25–55 years old age group showed a higher disease burden compared to other groups (Figure 4). Regions with high SDI had higher prevalence and DALYs (Supplementary Table S3) compared to other regions.

### Other neurological disorders

In 2019, other neurological disorders resulted in 4,263.39 (3,458.86, 5,174.14) DALYs in thousands (Table 1), which increased by 56.46% (41.8, 74.34) since 1990 (Figure 1). The burden on other neurological disorders was mainly seen in the 1–25 years old group (Figure 4). Both the age-standardized prevalence and DALY rates showed a weak positive correlation with the SDI weak (Supplementary Table S3).

### Tetanus

From 1990 to 2019, both the numbers and age-standardized rates of incidence, prevalence and deaths decreased significantly (Table 1), and the rank of the burden on tetanus moved down from the 5th to the 15th (Figure 1). Both the age-standardized prevalence and DALY rates showed a negative correlation with the SDI (all *p* < 0.001) (Supplementary Table S3). The burden of disease mainly impacted the early neonatal and late neonatal age groups (Figure 4).

### Down syndrome

The incidence, prevalence and deaths of down syndrome remained relatively stable from 1990 to 2019, but the number of DALYs decreased (Table 1). The burden of disease of Down syndrome peaked in the post neonatal group (Figure 4). Although age-standardized prevalence rate showed a strong positive correlation with SDI (*r* = 0.71, *p* < 0.001), the age-standardized DALY rate showed a moderate negative correlation with the SDI (*r* = −0.41, *p* < 0.001) (Supplementary Table S3).

### Multiple sclerosis

In 2019, multiple sclerosis resulted in 1,159.83 (1,001.18, 1,381.87) in thousands DALYs, which increased by 59.74% (46.63, 72.67) since 1990. The burden of multiple sclerosis for women was significantly higher than that of men (Table 1 and Figure 1). The burden of disease was higher in 45–60 years old group compared to other age groups (Figure 4). Both the age-standardized prevalence and DALY rates showed a strong positive correlation with the SDI (all *p* < 0.001) (Supplementary Table S3).

### Motor neuron disease

In 2019, the DALYs due to motor neuron disease was 1,034.61 (979.91, 1,085.40) in thousands (Table 1). The burden of motor neuron disease mainly impacted the 55–75 years old group (Figure 4). Same as for multiple sclerosis, both age-standardized prevalence and DALY rates showed a very strong positive correlation with the SDI (all *p* < 0.001) (Supplementary Table S3).

### Risk factors

Based on data of risk factors on the 18 neurological disorders from GDB 2019, it was observed that the DALYs and deaths of stroke were mainly attributed to high systolic blood pressure, with 55.54% (48.16, 62.03%) and 52.57% (44.43, 60.14%) attribution, respectively. Followed by high body-mass index, high fasting plasma glucose, and ambient particulate matter pollution (all with >15% attribution). Compared to 1990, their attribution all increased (Supplementary Table S4). Comparing the Joinpoint regression results of risk factors, it was observed that the attribution of high body-mass index to the age-standardized DALY rate showed an increasing trend in recent years (Annual Percent Change, APC = 0.25, *P* < 0.05) (Supplementary Figure S1).

The DALYs and deaths of neonatal encephalopathy due to birth asphyxia and trauma were mostly attributable to low birth weight and short gestation. Although the attribution of ambient particulate matter pollution was relatively low, DALYs and deaths due to this factor had an increasing trend from 1990 to 2019 (The maximum APC were 3.80 and 3.81, respectively, all *P* < 0.05) (Supplementary Figure S2).

In 2019, smoking contributed to 15.65% (10.10%, 20.99%) of total DALYs and 13.12% (8.15%, 17.96%) of total deaths of Alzheimer's disease and other dementias, which presented a downward trend when compared to 1990 (Supplementary Table S4). Attribution of metabolic risks (including high body-mass index and high fasting plasma glucose) had increased compared to 1990. Their contribution in 2019 was 20.86% (9.34, 35.52%) to DALYs and 20.66% (9.10, 35.24%) to deaths, which exceeded that of smoking. Of note, except for smoking, the disease burden attributable to high body-mass index and high fasting plasma glucose both showed an increasing trend (Supplementary Figure S3).

## Discussion

In this comprehensive and extensive analysis, we found that neurological disorders continue to contribute significantly to the total global disease burden in 2019, especially in cognitive-motor impairment. These 18 neurological disorders exhibit different disease burden profiles and temporal trends across regions and age groups. Notably, age-standardized DALY rates for these neurological disorders were associated with SDI, except for Alzheimer's disease and other dementias. Furthermore, we identified risk factors that contributed significantly to neurological conditions, such as high systolic blood pressure and high body-mass index.

In 2019, stroke, neonatal encephalopathy due to birth asphyxia and trauma, and migraine were ranked as the top three at the DALYs globally. The degree of disease burden regarding neurological disorders varied among the regions. For total neurological disease burden, the highest age-standardized DALY rates of neurological disorders were estimated for Western, Central, and Eastern Africa, most countries in East and Southeast Asia, whereas the lowest DALY rates were estimated for America, Europe and Oceania. For individual neurological disorders, 21 GBD regions ranked differently. For example, meningitis ranked low in many regions but ranked high in Eastern Sub-Saharan Africa, Oceania, South Asia, Southeast Asia, and Western Sub-Saharan Africa. This may be caused by underutilized interventions and preventive strategies, suggesting that it is necessary for the government of these regions to enhance policy implementation and prioritize the utilization of limited healthcare resources.

We noted significant age-specific and sex-specific patterns in the DALYs of many neurological disorders. The bulk of burden attributable to neonatal encephalopathy due to birth asphyxia and trauma occurred in early and late neonatal, and consequently results in the highest number of DALYs in the early neonatal group. Meningitis caused the most burden in post-neonatal and the 1–4 years old age group. DALYs due to neural tube defects mainly occurred in early neonatal, post-neonatal, and the 1–4 years old age groups. The burden caused by idiopathic epilepsy occurred mainly in 5–30 years old age group, while the burden caused by migraine peaked in the 15–34 years old age group. The burden of other neurological disorders gradually increased with advanced age. The rapid decline of global burden in adults older than 85 years old may be caused by competing mortality from other disorders with heavy burdens, such as cardiovascular disease and cancer. The higher burden (age-standardized DALYs, prevalence, deaths, incidence) of Parkinson's disease seen in males was consistent with previously reported global trends (2). The higher incidence of head and spinal injuries in males may be due to the propensity of traffic accidents. For migraine and multiple sclerosis, the higher age-standardized prevalence in females was consistent with GBD 2016 (1).

Although the overall burden of neurological disorders, as quantified by age-standardized rates of DALYs and deaths, have decreased from 1990 to 2019, the absolute numbers of DALYs and deaths had markedly increased. This indicated population growth and aging as the main drivers of increased burden. As an exception, communicable neurological disorders, including encephalitis, meningitis, and tetanus, decreased substantially in terms of absolute numbers and age-standardized rates of DALYs, prevalence, mortality, and incidence, which was consistent with previously observed results of overall global burden (1). The declining trend of communicable neurological disorders revealed the encouraging outcomes of promotion of vaccines and public health administration (6–10). The increase in prevalence and decrease of DALYs in neural tube defects and neonatal encephalopathy due to birth asphyxia and trauma could be the result of advances in medical technology. Policies and preventive interventions should be commended for their efforts to minimize the disease burden. However, they require to be strengthened if greater progress is to be achieved. Given that there is currently no available cure or effective treatment for most of the non-communicable neurological disorders, the absolute increase of cases deserves more attention.

In addition to population aging and growth, the substantial increase in the global burden of neurological disorders could be caused by increased exposure to risk factors. The burdens of individual neurological disorders attributable to various risk factors were different among people of different ages and sexes, which should be paid more attention and considered in preventive policies. Metabolic risks including high systolic blood pressure, high body-mass index and high fasting plasma glucose were important influences in stroke and Alzheimer's disease and other dementias with an upward trend, which was consistent with other studies (11, 12). Alarmingly recent studies reported a decreased level of hypertension awareness in the US population whose blood pressure has been controlled (13). Since 1980, the prevalence of obesity had doubled in over 70 countries (14), which may be caused by change in food composition and decreased levels of physical activity. The remarkable health loss attributable to the increasing prevalence of obesity and high fasting plasma glucose has become a stark reality. Therefore, appropriate and effective strategies are urgently required to decrease exposure to the risk factors. Increasing physical activity and maintaining a healthy diet could not only control the body-mass index, but also the fasting plasma glucose (15). Specifically, the government could provide incentives to increase healthy food production and use taxation to decrease unhealthy food consumption, as well as provide subsidies to increase level of physical activity (16).

The major strength of our study is the global assessment of the burden of major neurological disorders and identification of their potential risk factors based on the most up-to-date data. The findings could be valuable for policy-makers to develop evidence-based planning, establish priority-based cost-effective methods, minimize the modifiable risk factors, and reverse the upward trends. In addition, the age, sex, and region-specific information on the burden and attributable risk factors allowed policymakers to evaluate the effects of disease control programs and prioritize utilization of limited resources at a regional level.

Our study provided high-quality and comprehensive estimates of global neurological disorders burden, yet it is not free from limitations shared by all previous GBD estimates. First, the subtypes of individual neurological disorders, such as stroke, headache disorders, and dementia, were not shown in this study, which may have different epidemiological features and correspondingly, different intervention strategies. The sparse data after subdividing may lead to additional bias, which is the main challenge of disorders subdivision (3, 17). Second, data availability of many disorders is still the main limitation in burden estimates, especially in low-income and middle-income regions. Although covariates and other techniques have been used to obtain the best possible estimates, the barriers to data availability in those regions have to be eliminated to improve the robustness of estimates. Third, although we have completed a relatively comprehensive assessment for neurological disorders burden, some disorders such as rabies and neurocysticercosis were not included in our estimates, possibly resulting in the underestimation of the total burden due to neurological disorders (18). Fourth, although efforts have been made to minimize methodological differences and heterogeneity in case definitions, some variations among regions and countries may still lead to measurement error. Further standardization and improvement of the methods will allow us to more accurately estimate and compare the burden across geographical borders. Fifth, in many low-income and middle-income regions, cause-specific death rates mainly based on verbal autopsy data may be inaccurate as they are unable to identify cause of death in many neurological disorders (19).

## Conclusion

Although the trend of change in deaths and DALYs in communicable neurological disorders between 1990 and 2019 is encouraging, the total burden of neurological disorders is increasing with the growing and aging population. The trends of burden varied across geographical regions due to genetic factors, socioeconomic factors, sociodemographic factors, environmental factors, and local medical conditions, prompting more actions and efforts in the world, especially in economically deprived areas. Also, the government and policy-makers should take into account the variations of trends when designing preventive strategies and allocating resources.

## Data availability statement

Publicly available datasets were analyzed in this study. This data can be found here: GBD 2019 (http://ghdx.healthdata.org).

## Ethics statement

Ethical review and approval was not required for the study on human participants in accordance with the local legislation and institutional requirements. Written informed consent from the participants' legal guardian/next of kin was not required to participate in this study in accordance with the national legislation and the institutional requirements.

## Author contributions

FC and CD conceived the study and provided overall guidance. FC, YW, and XC prepared the first draft and finalized the manuscript based on comments from all other authors. FC, YW, CD, and XC had major roles in formulating the analysis using GBD 2019 methods. All authors contributed to the analysis and reviewed the manuscript.

## Funding

This work was supported by the Program for Excellent Talents in the First Affiliated Hospital of Fujian Medical University (Nos. YJCRC-B-KDZ2021 and YYXQN-DCY2021).

## Conflict of interest

The authors declare that the research was conducted in the absence of any commercial or financial relationships that could be construed as a potential conflict of interest.

## Publisher's note

All claims expressed in this article are solely those of the authors and do not necessarily represent those of their affiliated organizations, or those of the publisher, the editors and the reviewers. Any product that may be evaluated in this article, or claim that may be made by its manufacturer, is not guaranteed or endorsed by the publisher.
