# Supplementary material for: Global, regional, and national burden and attributable risk factors of neurological disorders: The Global Burden of Disease study 1990–2019
Source: Front Public Health. 2022 Nov 29;10:952161. doi: 10.3389/fpubh.2022.952161 (PMC9745318; doi:10.3389/fpubh.2022.952161)
Supplement: Supplementary file 1 [file Data_Sheet_1.docx]

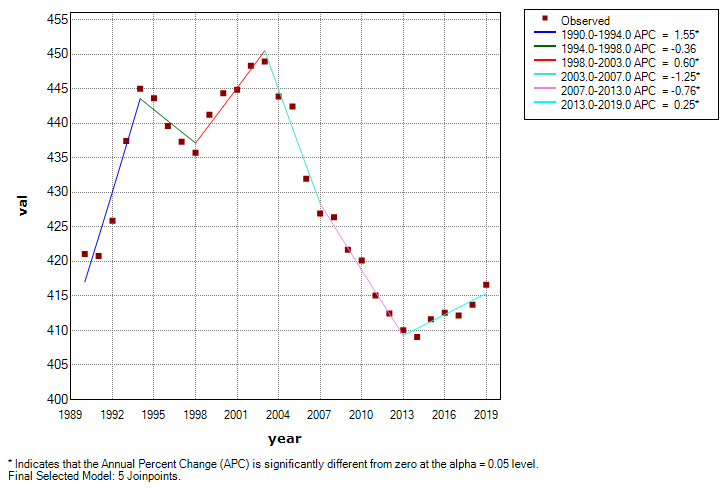
**Figure S1** Temporal trends in DALYs of global stroke attributable to high body-mass index from 1990 to 2019 for all ages and both sexes.


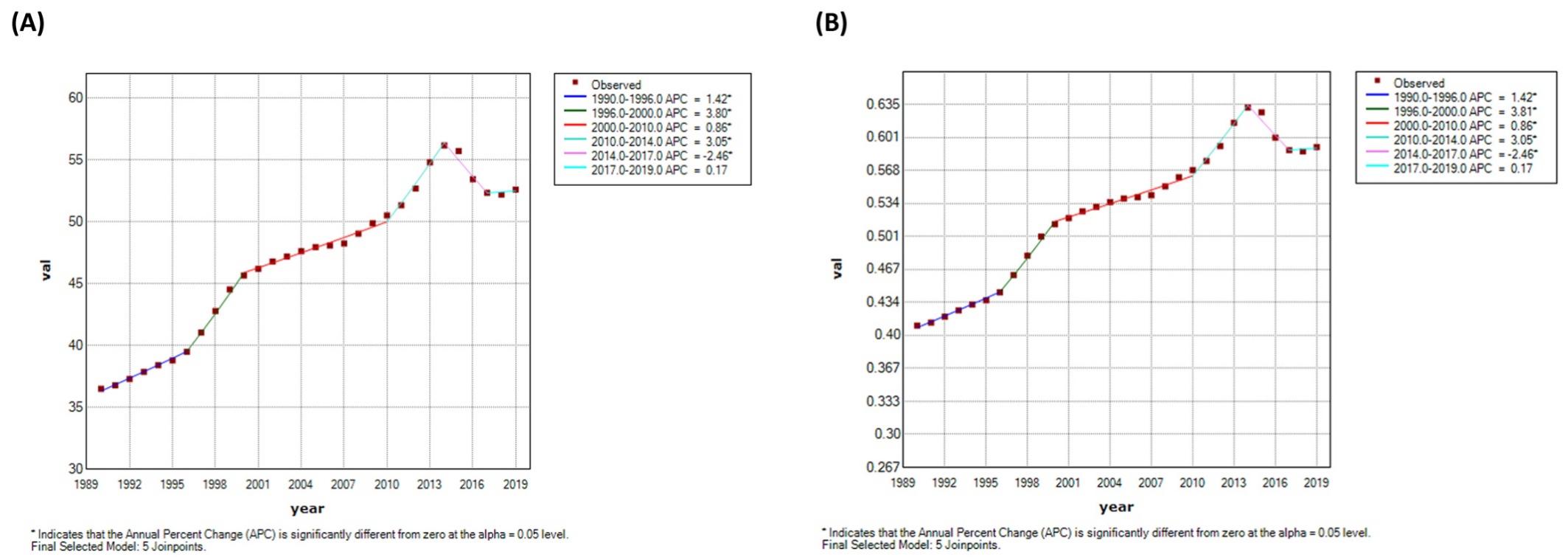


**Figure S2** Temporal trends of global neonatal encephalopathy due to birth asphyxia and trauma attributable to ambient particulate matter pollution from 1990 to 2019 for all ages and both sexes. (A) DALYs attributable to ambient particulate matter pollution; (B) Deaths attributable to ambient particulate matter pollution.


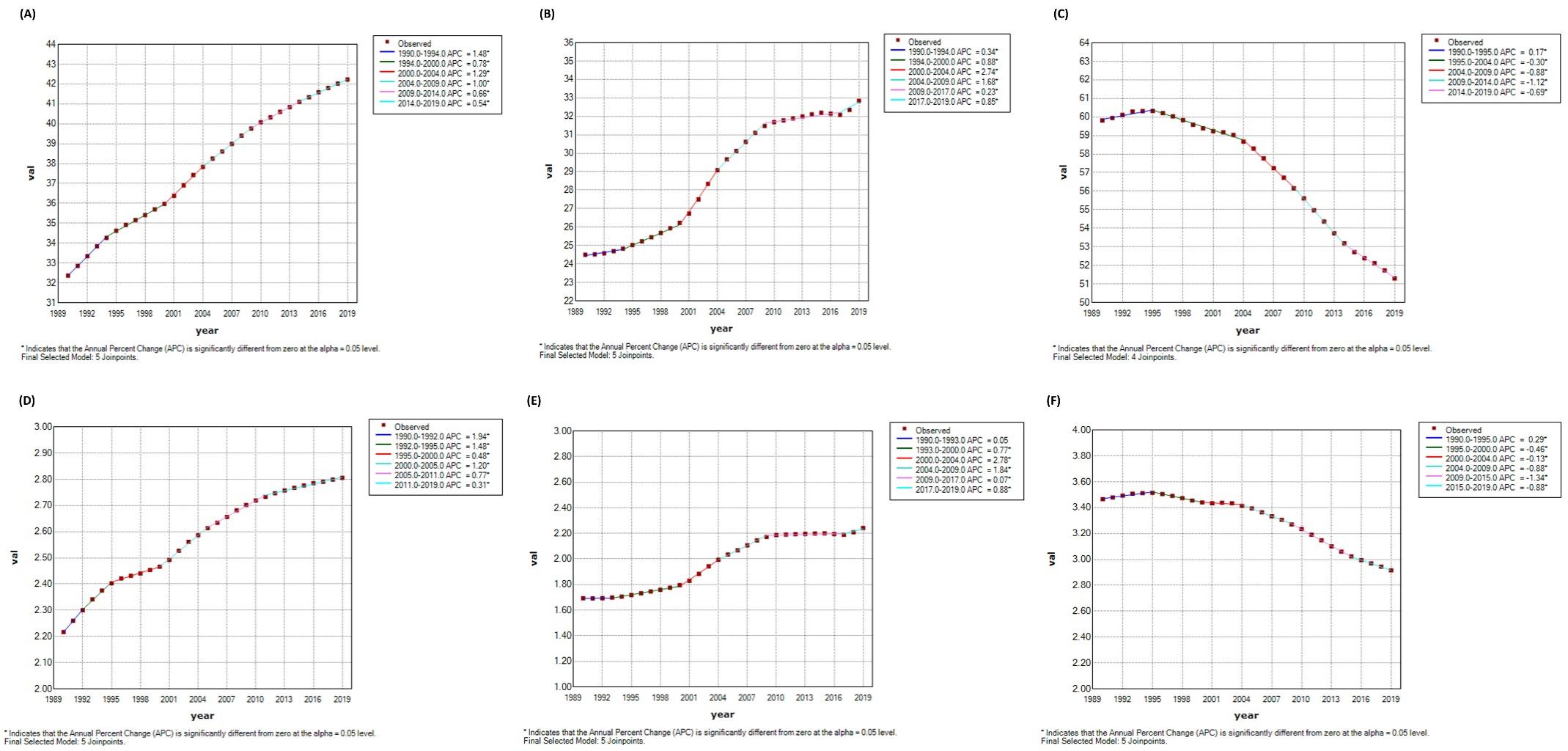


**Figure S3** Temporal trends of global Alzheimer's disease and other dementias attributable to risk factors from 1990 to 2019 for all ages and both sexes. (A) DALYs attributable to high body-mass index; (B) DALYs attributable to high fasting plasma glucose; (C) DALYs attributable to smoking; (D)Deaths attributable to high body-mass index; (E) Deaths attributable to high fasting plasma glucose; (F) Deaths attributable to smoking.
